# IgG4-Related Disease: A Multispecialty Condition

**DOI:** 10.1155/2014/723493

**Published:** 2014-11-24

**Authors:** Iuri Usêda Santana, Emanuela Pimenta da Fonseca, Mittermayer Barreto Santiago

**Affiliations:** ^1^Serviço de Clínica Médica do Hospital Geral Roberto Santos, Rua Direta do Saboeiro s/n, Cabula, 41180-900 Salvador, BA, Brazil; ^2^Serviços Especializados em Reumatologia (SER) da Bahia, Rua Conde Filho 117, Graça, 40150-150 Salvador, BA, Brazil

## Abstract

IgG4-related disease (IgG4-RD) is a recently recognized group of conditions, characterized by tumor-like swelling of involved organs, lymphoplasmacytic infiltrate rich in IgG4-positive plasma cells, variable degrees of fibrosis, and elevated serum IgG4 concentrations. Currently IgG4-RD is recognized as a systemic condition that can affect several organs and tissues. Herein we report the case of a 34-year-old male patient who was admitted to our hospital with diffuse abdominal pain, weight loss, and painful stiffness in his neck. He had a history of tumoral mass of the left maxillary region, right palpebral ptosis with protrusion of the eyeball, and chronic dry cough for about 6 years. Laboratory tests revealed polyclonal hypergammaglobulinemia and increased serum IgG4 levels. Immunohistochemical staining of the maxillary biopsy was compatible with IgG4-RD. He had an excellent response to corticosteroid therapy. This case highlights that IgG4-RD should be included in the differential diagnosis with multisystem diseases.

## 1. Introduction

IgG4-related disease (IgG4-RD) is a recently recognized fibroinflammatory group of disorders of unknown etiology, characterized by tumor-like swelling of the involved organs, tissue lymphoplasmacytic infiltrate rich in IgG4-positive plasma cells, variable degrees of fibrosis, and, often, elevated serum IgG4 concentrations. It was recognized as a systemic condition in 2003, in patients with autoimmune pancreatitis with extrapancreatic manifestations [1]. In this context it seems to be analogous to sarcoidosis, another disease in which similar histopathological findings are observed in various affected organs.

Several clinical conditions encompass the IgG4-RD: Type 1 autoimmune pancreatitis; sclerosing cholangitis; dacryoadenitis and sclerosing sialadenitis; inflammatory orbital pseudotumor; idiopathic retroperitoneal fibrosis; chronic sclerosing aortitis; Riedel's thyroiditis; IgG4-related interstitial pneumonitis, pulmonary inflammatory pseudotumors, tubulointerstitial nephritis, hypophysitis, and pachymeningitis and many other associations are likely to be revealed [1–7].

The aim of the present report was to illustrate a case, in which diagnosis was delayed for years before IgG4-RD was recognized, drawing attention to the multidisciplinary nature of this condition and the necessity for suspecting its presence in patients with unspecific signs and symptoms, particularly when associated with histological findings of “chronic inflammatory process.”

## 2. Case Report

A 34-year-old man was admitted to our hospital with a 4-month history of diffuse abdominal pain and body weight loss, progressing in 3 months to muscle weakness, abdominal distension, nausea, vomiting, and stiff painful swelling of his neck. His previous history included a fluctuating tumoral mass in the left maxillary region for the last six years, causing mild dysphagia and limiting movement of the temporomandibular joint. The pathological examination was consistent with a chronic inflammatory process. Later on he presented right palpebral ptosis with protrusion of the eyeball and chronic dry cough, with transbronchial biopsy examination revealing chronic pneumonitis and irregular septal fibrosis.

On examination he was afebrile, with blood pressure 120/80 mmHg, with pale mucous membranes, anterior neck swelling and stiffness with local hyperemia, without palpable lymph nodes, reduced pulmonary expansion, diffuse abdominal pain, and rigidity on palpation. The remainder of the physical examination was unremarkable.

On admission laboratory tests revealed the following: hemoglobin 9.0 g/dL, white blood cell count 21,390/mm^3^, platelet count 808,000/mm^3^, total bilirubin 2.89 mg/dL (direct bilirubin 2.26 mg/dL), calcium 8.2 mg/dL, creatinine 0.4 mg/dL, aspartate transaminase 25.7 mg/dL, alanine transaminase 16.6 mg/dL, and alkaline phosphatase 417 mg/dL. Search for antinuclear antibodies (ANA), anti-SSA/Ro, and ANCA showed negative results. Serum protein electrophoresis and immunofixation showed polyclonal hypergammaglobulinemia. Serum IgG4 was 264 mg/dL (reference value ≤140 mg/dL). Ultrasonography of the neck showed soft tissues swelling. Abdominal ultrasonography was normal.

The patient developed daily fever, asthenia, diarrhea, profuse coughing, and intense dyspnea. Chest radiography revealed bilateral parenchymal infiltration, multiple pulmonary nodules, and consolidation in the lower right hemithorax. As the patient's clinical condition was not improving, despite the use of broad spectrum antibiotics (piperacillin/tazobactam, meropenem) and mechanical ventilation, pulse therapy with methylprednisolone 1 g was started and used for three days, with considerable clinical and radiological improvement. After this, prednisone 50 mg/day was initiated and he was discharged in good general condition. After two-year follow-up, he has been well on lower dose (5 mg/day) of prednisone. In the last few months, after abrupt voluntary discontinuation of his medication, there was a mild relapse of the disease which remitted with the reintroduction of 20 mg/day of prednisone. The patient remains asymptomatic with low doses of steroids, maintaining complete resolution of respiratory, eye, and neck symptoms which were assumed to be manifestations of the same disease.

**Figure 1:**
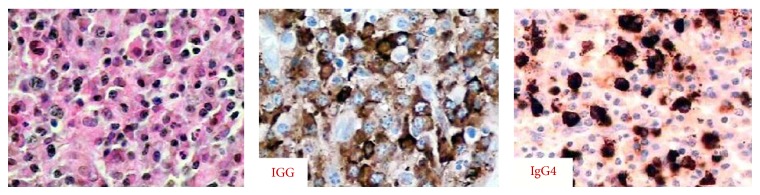
Histological sample from maxillary biopsy with immunohistochemical staining showing infiltrate rich in plasma cells with increased proportion of positive IgG4 cells.

A reanalysis (with immunohistochemical staining) of the histological sample from previous maxillary biopsy revealed chronic inflammatory infiltrate rich in plasma cells with areas of fibrosis and erosion. Immunohistochemistry showed mixed population of B and T lymphocytes and an increased amount of positive IgG4 cells (up to 55 IgG4-positive plasma cell per high power field; IgG4/IgG ratio of 45%) (Figure 1).

## 3. Discussion

The pathogenesis of IgG4-RD is poorly understood; however some findings are consistent with multiple immune-mediated mechanisms [8]: class II histocompatibility antigen genotype; autoantibodies to lactoferrin and carbonic anhydrase II; possible molecular mimicry involving* Helicobacter pylori*; immune complex deposition in some affected organs; increased levels of Th2 cytokines, T regulatory cells, interleukin-10, and transforming growth factor; evidences of allergic response with peripheral eosinophilia.

The overall epidemiology of the disease remains largely undefined. IgG4-RD is typically described in middle-aged and older men, mostly in autoimmune pancreatitis; the disease prototype and patients usually have a history of atopic disease [9–11].

Multiorgan disease can be evident at diagnosis, but it usually evolves metachronously over a variable time. Estimates of the relative frequency of different manifestations depend upon the perspective of the index illness being analyzed.

Sometimes IgG4-RD is a diagnostic challenge, because it has multiple clinical manifestations and a wide spectrum of differential diagnosis. The confirmation of diagnosis is based on histopathological findings of the affected organ with immunohistochemical staining. The serum IgG4 level should be measured as it may strengthen the diagnostic hypothesis, although about 30% of patients may have normal serum IgG4 concentrations, despite classic histopathological findings of IgG4-RD [12].

This report well illustrates a common clinical situation of delayed diagnosis of IgG4-RD. Many reports of inflammatory pseudotumor in different sites may be examples of undiagnosed IgG4-RD [13, 14]. In the present case, this condition should have been suspected after the histopathological results indicating “chronic inflammatory processes” in the tissue specimens. However our patient spent six years before the diagnosis was defined. His clinical findings included an inflammatory pseudotumor of the maxillary region and proptosis, probably secondary to a retro-orbital pseudotumor. Curiously enough, both conditions had periods of spontaneous remission and relapse. In addition, the patient reported a chronic dry cough with transbronchial biopsy revealing chronic pneumonitis and irregular septal fibrosis. Another unique feature of his disease was the presence of neck stiffness. Such atypical manifestation was probably secondary to idiopathic cervical fibrosis regarded as a new member of IgG4-RD [15]. Apart from the histopathological features, his excellent short and long-term response to the therapy with glucocorticoids, remaining asymptomatic with low doses of steroids, reinforced the diagnosis of IgG4-RD.

This case highlights the need to recognize IgG4-RD as an important differential diagnosis with multisystem diseases as the treatment may lead to remission and prevent significant morbidity and mortality.

## References

[1] Kamisawa T., Funata N., Hayashi Y., Eishi Y., Koike M., Tsuruta K., Okamoto A., Egawa N., Nakajima H. (2003). A new clinicopathological entity of IgG4-related autoimmune disease. *Journal of Gastroenterology*.

[2] Stone J. R. (2011). Aortitis, periaortitis, and retroperitoneal fibrosis, as manifestations of IgG4-related systemic disease. *Current Opinion in Rheumatology*.

[3] Dahlgren M., Khosroshahi A., Nielsen G. P., Deshpande V., Stone J. H. (2010). Riedel's thyroiditis and multifocal fibrosclerosis are part of the IgG4-related systemic disease spectrum. *Arthritis Care and Research*.

[4] Dechoux S., Arrivé L. (2011). IgG4-related sclerosing disease: autoimmune pancreatitis (AIP) and IgG4-related cholangitis. *Clinics and Research in Hepatology and Gastroenterology*.

[5] Teichman J. C., Wu A. Y., El-Shinnawy I., Harvey J. T. (2012). A case of orbital involvement in IgG4-related disease. *Orbit*.

[6] Vlachou P. A., Khalili K., Jang H.-J., Fischer S., Hirschfield G. M., Kim T. K. (2011). IgG4-related sclerosing disease: autoimmune pancreatitis and extrapancreatic manifestations. *Radiographics*.

[7] Cornell L. D. (2012). IgG4-related kidney disease. *Current Opinion in Nephrology and Hypertension*.

[8] Stone J. H., Zen Y., Deshpande V. (2012). Mechanisms of disease: IgG4-related disease. *The New England Journal of Medicine*.

[9] Raina A., Yadav D., Krasinskas A. M., McGrath K. M., Khalid A., Sanders M., Whitcomb D. C., Slivka A. (2009). Evaluation and management of autoimmune pancreatitis: experience at a large us center. *American Journal of Gastroenterology*.

[10] Nishimori I., Tamakoshi A., Otsuki M. (2007). Prevalence of autoimmune pancreatitis in Japan from a nationwide survey in 2002. *Journal of Gastroenterology*.

[11] Kamisawa T., Anjiki H., Egawa N., Kubota N. (2009). Allergic manifestations in autoimmune pancreatitis. *European Journal of Gastroenterology and Hepatology*.

[12] Sah R. P., Chari S. T. (2011). Serologic issues in IgG4-related systemic disease and autoimmune pancreatitis. *Current Opinion in Rheumatology*.

[13] Leitão B., Machado F., Soares F., Souza H., Queiroz A. C., Santiago M. B. (2009). Myocardial inflammatory pseudotumor and multiple thromboses as a manifestation of Behcet disease. *Journal of Clinical Rheumatology*.

[14] Andrade D. M. S., Martins S. J., Paz O., Cardozo J. B., Novaes A. E., Santiago M. B. (2006). Inflammatory pseudotumor: a diagnostic dilemma. *European Journal of Internal Medicine*.

[15] Cheuk W., Tam F. K. Y., Chan A. N. H., Luk I. S. C., Yuen A. P. W., Chan W.-K., Hung T. C. W., Chan J. K. C. (2010). Idiopathic cervical fibrosis—a new member of IgG4-related sclerosing diseases: report of 4 cases, 1 complicated by composite lymphoma. *The American Journal of Surgical Pathology*.

